# Transmission electron microscopy characterization of fluorescently labelled amyloid β 1-40 and α-synuclein aggregates

**DOI:** 10.1186/1472-6750-11-125

**Published:** 2011-12-19

**Authors:** Valerie L Anderson, Watt W Webb

**Affiliations:** 1 School of Applied and Engineering Physics, Cornell University, Ithaca, NY, USA

## Abstract

**Background:**

Fluorescent tags, including small organic molecules and fluorescent proteins, enable the localization of protein molecules in biomedical research experiments. However, the use of these labels may interfere with the formation of larger-scale protein structures such as amyloid aggregates. Therefore, we investigate the effects of some commonly used fluorescent tags on the morphologies of fibrils grown from the Alzheimer's disease-associated peptide Amyloid β 1-40 (Aβ40) and the Parkinson's disease-associated protein α-synuclein (αS).

**Results:**

Using transmission electron microscopy (TEM), we verify that N-terminal labeling of Aβ40 with AMCA, TAMRA, and Hilyte-Fluor 488 tags does not prevent the formation of protofibrils and amyloid fibrils of various widths. We also measure the two-photon action cross-section of Aβ40 labelled with Hilyte Fluor 488 and demonstrate that this tag is suitable for use with two-photon fluorescence techniques. Similarly, we find that Alexa Fluor 488 labelling of αS variant proteins near either the N or C terminus (position 9 or 130) does not interfere with the formation of amyloid and other types of αS fibrils. We also present TEM images of fibrils grown from αS C-terminally labelled with enhanced green fluorescent protein (EGFP). Near neutral pH, two types of αS-EGFP fibrils are observed via TEM, while denaturation of the EGFP tag leads to the formation of additional species.

**Conclusions:**

We demonstrate that several small extrinsic fluorescent tags are compatible with studies of amyloid protein aggregation. However, although fibrils can be grown from αS labelled with EGFP, the conformation of the fluorescent protein tag affects the observed aggregate morphologies. Thus, our results should assist researchers with label selection and optimization of solution conditions for aggregation studies involving fluorescence techniques.

## Background

Fluorescent tags are commonly used to monitor proteins and peptides in microscopy and spectroscopy experiments [1-3]. However, incorporation of these labels may affect protein structure or block protein-ligand interactions; therefore it is important to verify that specific tags and labelling locations are suitable for a particular application. In the context of amyloid aggregation studies, in which proteins or peptides associate to form various oligomeric structures, it is necessary to investigate potential perturbations of the aggregation reaction due to the presence of the label. In particular, because multiple fibril types may be grown from one protein or peptide [4], it is essential to ensure that incorporation of a label is compatible with multiple aggregation pathways.

In this Paper, we present TEM and other characterizations of fluorescently labelled Aβ40 peptide and αS protein. Aβ is associated with Alzheimer's disease, while αS is linked to Parkinson's disease; therefore fluorescently labelled αS/Aβ constructs may be useful for understanding the initiation and progression of these common human neurodegenerative disorders. Indeed, fluorescently-labelled Aβ and αS constructs have been used in numerous studies of protein interactions, trafficking, and degradation, as well as in investigations of structural changes linked to amyloid aggregation (see articles referenced in [5,6]). However, relatively few researchers have examined the effects of these fluorescent tags on Aβ/αS aggregate morphologies [5,7-12].

High-resolution imaging techniques, including TEM and atomic force microscopy, enable identification and classification of aggregates, which may include protofibrils, amyloid fibrils containing varying numbers of strands, and amorphous aggregates. In contrast, methods used to quantify fibril production, such as thioflavin-T binding, light scattering, Fourier transform infrared spectroscopy, and circular dichroism spectroscopy, cannot discriminate among different types of β-sheet rich species [13,14]. Nevertheless, TEM imaging provides no quantitative information about aggregation kinetics or about the concentrations of the observed fibrils. In addition, rare species or aggregates that do not stick to the TEM grids may not be detected. Therefore, TEM can confirm the presence of a particular type of aggregate, but it cannot prove that a type is disallowed.

We examine Aβ40, peptides tagged with three extrinsic fluorophores (AMCA, TAMRA, and Hilyte Fluor 488). These labels were selected because their emission peaks are reasonably well-separated, making them potentially useful for multi-channel imaging or fluorescence cross-correlation spectroscopy applications. In addition, peptides tagged with these dyes are readily available from commercial sources.

For αS, we compare small organic dye (Alexa Fluor 488) with fluorescent protein (EGFP) labelling. Alexa Fluor 488, EGFP, and Hilyte Fluor 488 have similar excitation and emission spectra, and therefore are compatible with similar optical systems. In addition, investigations of the effects of fluorescent protein tags are particularly important given the high potential value of these tags for *in vivo *and cell-based experiments [11]. However, the large size of most fluorescent proteins (~29 kDa) compared to αS (14.5 kDa), as well as the potential environmental sensitivity of fluorescent protein tags, raises questions regarding the suitability of αS-fluorescent protein constructs for aggregation studies [9,15].

Our TEM images show that several extrinsic fluorescent labels do not preclude the growth of multiple fibril varieties for Aβ40 and αS. In contrast, we observe two distinct types of rigid aggregates when αS-EGFP solutions are incubated near physiological pH. Moreover, disruption of the EGFP tag results in the growth of additional species. Therefore, although the fluorescent protein label does not prevent aggregation of the αS-EGFP construct, the fibrillization pathway is affected by the conformation of the EGFP tag. We believe that our images will provide a starting point to assist researchers in fluorophore selection for aggregation studies, although additional characterizations will be necessary for some applications.

## Results

### Extrinsic Dye Labelled Aβ40 Aggregates

**Figure 1:**
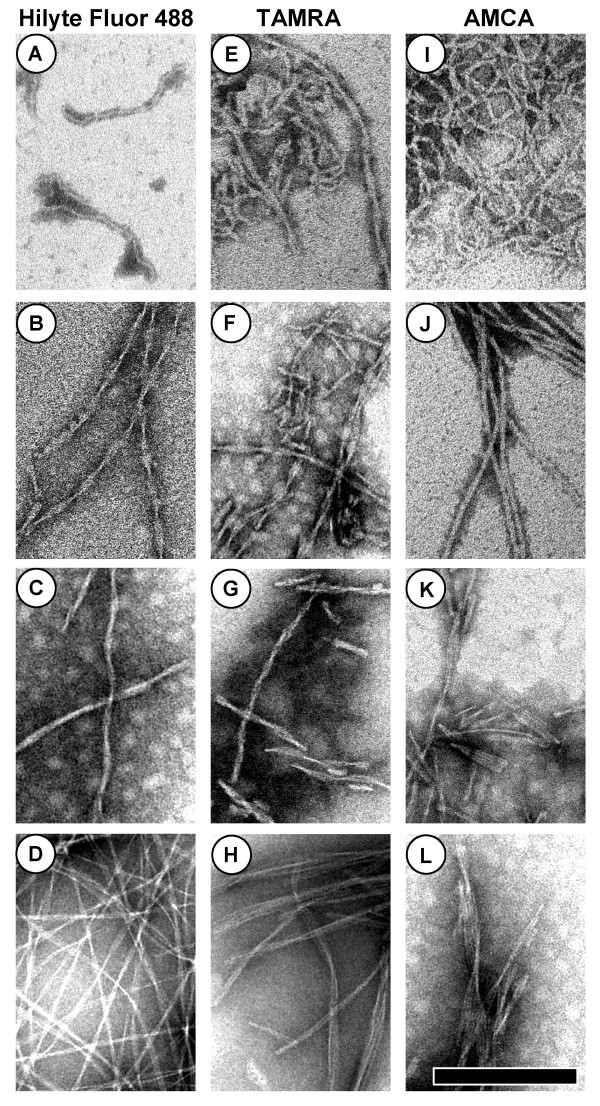
**TEM images of aggregates grown from Aβ40 labelled with three extrinsic fluorescent dyes**. Each column corresponds to one fluorophore. The scale bar is 200 nm wide and all images are shown at the same magnification. Unless otherwise noted, the samples were incubated at 22°C under quiescent conditions. The protein concentrations, incubation times *t*, and other solution conditions are as follows: (A) 200 μM Hilyte Fluor 488-Aβ40 in water, *t *= 7 days. (B) 15 μM Hilyte Fluor 488-Aβ40 in 100 mM pH 7 NaPhos buffer, *t = *8 weeks. (C) 10 μM Hilyte Fluor 488-Aβ40 in 50 mM pH 6 NaPhos, *t = *4 weeks. (D) 10 μM Hilyte Fluor 488-Aβ40 in 50 mM, pH 7 NaPhos with 5% TFE, *t = *10 weeks. (E) 25 μM TAMRA-Aβ40 incubated at 37°C with shaking in 50 mM pH 7 NaPhos with 10% TFE, *t *= 2 weeks. (F) 50 μM TAMRA-Aβ40 in 100 mM pH 7 NaPhos, *t = *8 weeks. (G) 73 μM TAMRA-Aβ40 in 50 mM pH 7 NaPhos, *t *= 15 weeks. (H) 50 μM TAMRA-Aβ40 in 50 mM pH 6 NaPhos, *t *= 20 weeks. (I) 25 μM AMCA-Aβ40 incubated at 37°C with shaking in 50 mM pH 7 NaPhos containing 10% TFE, *t *= 2 weeks. (J) 25 μM AMCA-Aβ40, shaken overnight at 37°C, followed by incubation at 22°C under quiescent conditions for 5 weeks. (K) 20 μM AMCA-Aβ40 in 50 mM, pH 7 NaPhos with 100 mM NaCl, *t *= 4 weeks. (L) 20 μM AMCA-Aβ40 in 50 mM, pH 7 NaPhos, *t *= 4 weeks.

Figure 1 shows columns of TEM images of aggregates grown from Hilyte Fluor 488-, TAMRA-, and AMCA-Aβ40. Various fibril types were apparent in these samples, including thin flexible "protofibrils" (Figure 1A,E-F,I) and rigid fibrils of various widths (Figure 1B-D,1E-H,J-L). Thus, labelling with these small organic fluorophores does not prevent the formation of many types of amyloid aggregates. In general, samples with higher concentrations of peptide and lower ionic strengths were more likely to generate protofibrils. However, details of the solution preparations caused variations in fibril types and morphologies were not wholly reproducible.

For the TAMRA and Hilyte Fluor 488 labelled Aβ40 samples shown in Figure 1, the solutions initially appeared uniformly fluorescent when viewed by eye (AMCA emits in the UV, and so the AMCA-Aβ40 samples appear transparent). After incubation in aggregation-promoting conditions, fluorescent clumps were apparent in the bottom of these sample tubes, while the supernatants of these solutions became more transparent, suggesting that the TAMRA and Hilyte Fluor 488 tags were incorporated into the observed aggregates. Furthermore, the labelling efficiencies were high for the synthetic Aβ40 peptides we examined (see Methods), and similar images were obtained for all three fluorophores; therefore the observed fibrils likely contained tagged peptides.

**Figure 2:**
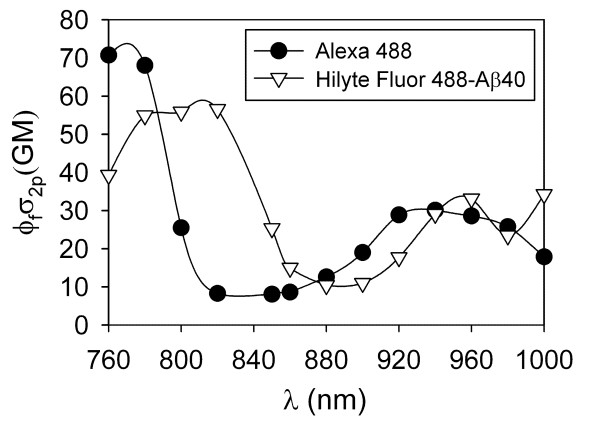
**Two-photon action cross-section of Hilyte Fluor 488 labelled Aβ40**. The two-photon action cross-section (ϕ_f_σ_2p_) of Hilyte Fluor 488 labelled Aβ40 is compared to that for Alexa Fluor 488 free dye. The units for ϕ_f_σ_2p_ are Goeppert-Mayer (GM), where 1 GM = 10^-50^ cm^4^ s photon^-1^.

Potential applications of fluorescently labelled peptides include two-photon imaging and two-photon fluorescence correlation spectroscopy. The two-photon action cross-sections of AMCA and TAMRA are sufficient for these applications [16-18]. However, the cross-section for Hilyte Fluor 488, which is an analogue of Alexa Fluor 488 has not been previously determined, as far as we know. Therefore, we measured the two-photon action cross-section for Hilyte Fluor 488-Aβ40, and compared this curve to the free Alexa Fluor 488 dye cross-section (Figure 2). We find that Hilyte Fluor 488 is a good two-photon probe when excited at ~760-850 nm and ~940-1000 nm, although its cross-section does not coincide exactly with the Alexa Fluor 488 spectrum.

### Alexa-488-αS Aggregates

**Figure 3:**
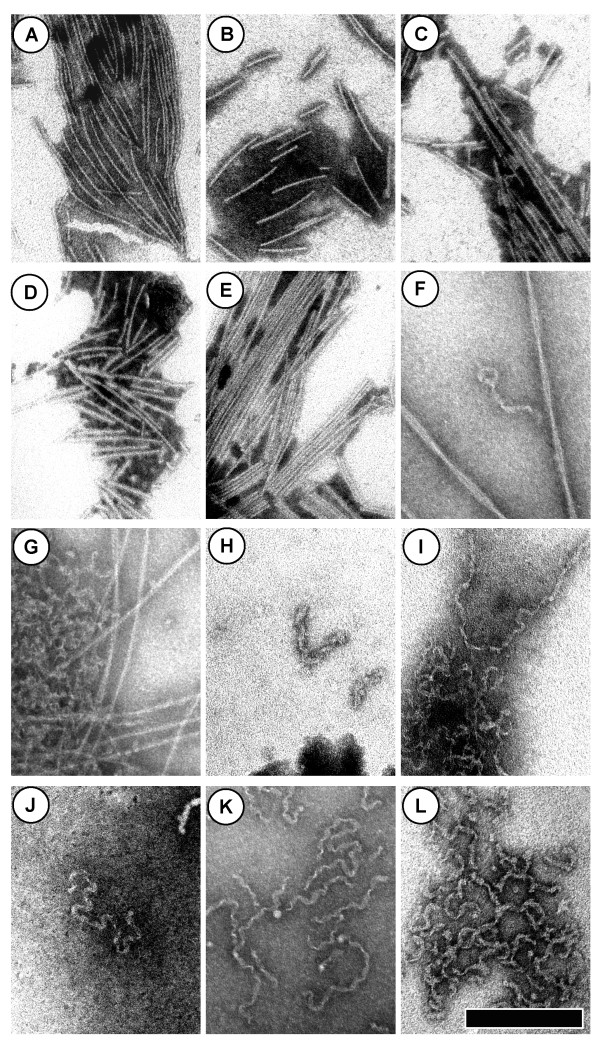
**TEM images of aggregates grown from αS variants labelled with Alexa Fluor 488**. The scale bar is 200 nm wide and all images are shown at the same magnification. All samples contained 10 mM pH 7.5 NaPhos, and unless otherwise specified, the solutions were incubated at 37°C with shaking. The protein concentrations, incubation times *t*, and other solution conditions are as follows: (A) 43 μM WT/E130C-Al488 αS with 150 mM NaCl, *t = *1 week. (B) 43 μM WT/S9C-Al488 αS with 150 mM NaCl and 5% TFE, *t = *1 week. (C) 50 μM A30P/E130C-Al488 αS with 150 mM NaCl, *t = *2 weeks. (D) 50 μM A53T/S9C-Al488 αS with 150 mM NaCl, *t = *2 weeks. (E) 50 μM A30P/E130C-Al488 αS with 150 mM NaCl and 5% TFE, *t = *2 weeks. (F-G) 50 μM A30P/E130C-Al488 αS with 5% TFE, *t *= 2 weeks. (H) 43 μM WT/S9C-Al488 αS with150 mM NaCl and 15% TFE, incubated at 22°C under quiescent conditions, *t *= 5 weeks. (I) 50 μM WT/E130C-Al488 αS with 150 mM NaCl and 5% TFE, incubated at 22°C under quiescent conditions, *t *= 2 weeks. (J) 50 μM WT/E130C-Al488 αS with 5% TFE, incubated at 22°C under quiescent conditions, *t *= 1 week. (K) 50 μM A30P/S9C-Al488 αS with 10% TFE, *t *= 2 weeks. (L) 50 μM A30P/E130C-Al488 αS, *t *= 2 weeks.

When aggregates grown from αS variant proteins labelled with Alexa Fluor 488 are imaged using TEM, we detect both rigid fibrils (Figure 3A-G) and flexible "TFE fibrils" (Figure 3F-L), which are similar to species previously observed for unlabelled αS in solutions containing TFE or detergents [19,20]. The rigid fibrils are most frequently narrow (~4-5 nm width) and straight, but may also appear twisted and thicker, with widths ranging from 10-20 nm. The "TFE fibrils" are typically ~10-13 nm wide. As was observed for the Aβ40 peptides, fluorescent aggregates grown from Alexa Fluor 488 labeled αS variant proteins were typically visible by eye after incubation.

For human wild-type (WT) αS, N-terminal (position 9) and C terminal (position 130) labelling is compatible with growth of both fibrils types (Figure 3A-B, 3H-J). We also investigated fluorescent aggregates grown from two Parkinson's disease-associated αS variants, A30P and A53T αS. For A30P αS, both N terminal and C terminal labels were examined (Figure 3C,E,F,G,K), while for A53T, we examined only N terminal labelling (Figure 3D). Although we do not investigate a wide range of solution conditions for the variants, we do observe fluorescently labelled aggregates for both mutants. Therefore, it is likely that both C terminal and N terminal labels may be employed for multiple αS variants.

Note that our images do not enable direct comparisons of aggregation properties of proteins labelled at the C vs. N termini because of variability in solution conditions and incubation times, as well as the possible presence of oligomeric species in the stock solutions. Therefore, we are only able to observe that certain structures are not precluded by the label, but cannot determine whether a label or labelling location may tend to favour a particular type of aggregate. More detailed studies are necessary to determine whether labelling has any subtle effects on aggregation pathway selection.

### EGFP-αS Aggregates

**Figure 4:**
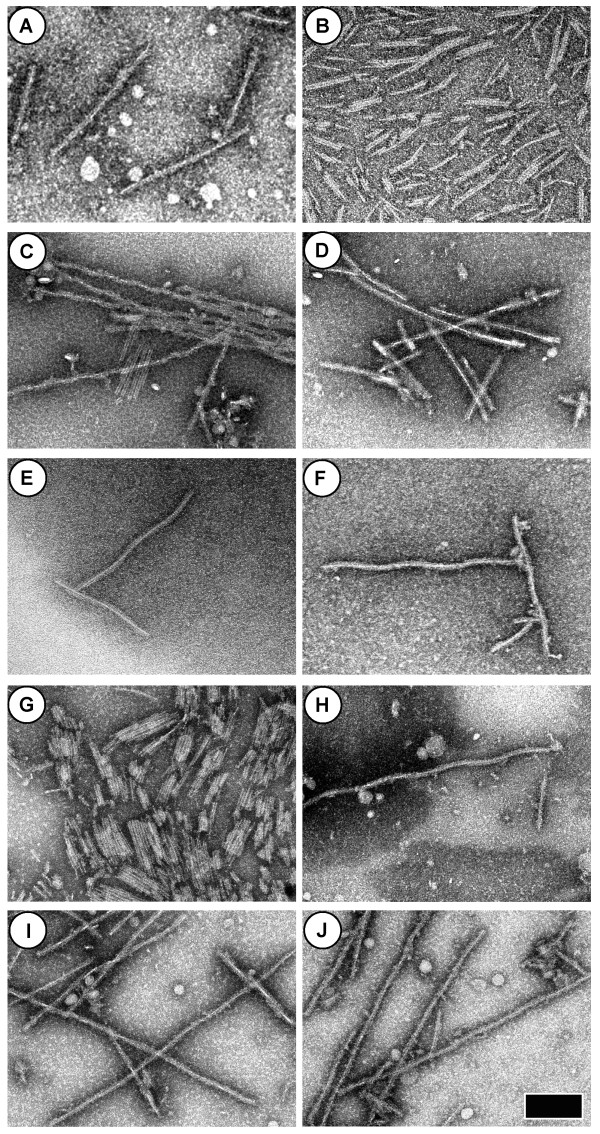
**TEM images of aggregates grown from seeded or unfiltered αS-EGFP stocks**. The scale bar is 200 nm wide and all images are shown at the same magnification. Unless otherwise specified, all solutions contained PBS buffer (10 mM pH 7.5 NaPhos, 150 mM NaCl) and the samples were incubated with shaking at 37°C. The protein concentrations, incubation times *t*, and other solution conditions are as follows: (A-B) 75 μM unfiltered αS-EGFP, *t *= 3 weeks. (C) 150 μM unfiltered αS-EGFP, *t *= 3 weeks. (D) 35 μM unfiltered αS-EGFP in pH 7.4 Tris buffer with 100 mM NaCl, *t *= 3 weeks. (E-G) 30 μM filtered αS-EGFP plus ~4 μM unfiltered αS-EGFP "seed" in pH 7.4 Tris with 100 mM NaCl, *t *= 4 weeks. (H) 20 μM unfiltered αS-EGFP in pH 7.4 Tris with 100 mM NaCl, *t *= 4 weeks. (I-J) 150 μM filtered αS-EGFP plus ~ 8 μM unfiltered αS-EGFP "seed", *t *= 4 weeks.

We observed fibrils when samples containing 75 μM and 150 μM αS-EGFP in PBS were prepared using unfiltered αS-EGFP stock solutions (Figure 4A-C). However, we were unable to detect fibrils via TEM when protein stocks were filtered (100 kDa cut-off) prior to incubation when all other solution conditions, including incubation time and protein concentration as measured by UV absorbance at 488 nm, were held constant. Similar results were obtained for αS-EGFP in Tris buffer with 100 mM NaCl; we observed fibrils in a 35 μM unfiltered sample (Figure 4D), but were unable to find fibrils in a sample prepared using a filtered protein stock and identical solution conditions and protein concentration. Interestingly, the addition of a small amount (~4 μM out of 34 μM total) of dialyzed, unfiltered protein to samples prepared using filtered protein resulted in the formation of αS-EGFP fibrils (Figure 4E-G). Although TEM imaging is not a quantitative technique, these preliminary results suggest that "seeding" samples with unfiltered or pre-aggregated material may promote fibril formation. However, additional experiments must be done to verify this result.

Additional images of αS-EGFP fibrils gown from seeded or unfiltered solutions at pH ~7.5 in various buffer conditions are shown in Figure 4H-J. The αS-EGFP fibrils appear to have a thin, straight core (~5-7 nm in diameter), around which winds a somewhat indistinct or blurry helix. The total fibril diameter is ~22 nm, and the helical period is variable, ranging from ~140 nm to over 300 nm. In some samples, shorter, untwisted, multi-stranded rigid fibrils were also observed (Figure 4B,G).

When αS-EGFP solutions containing fibrils were examined by eye, they appeared uniformly fluorescent, unlike the extrinsic fluorophore-labelled αS samples in which fluorescent aggregated material was clearly visible at the bottom of the tubes. This may be a result of αS-EGFP fibrils remaining suspended in solution, or the fibril fraction may be a minor component of the sample. Alternatively, the EGFP tag may be quenched or altered in the αS-EGFP fibrils [11].

**Figure 5:**
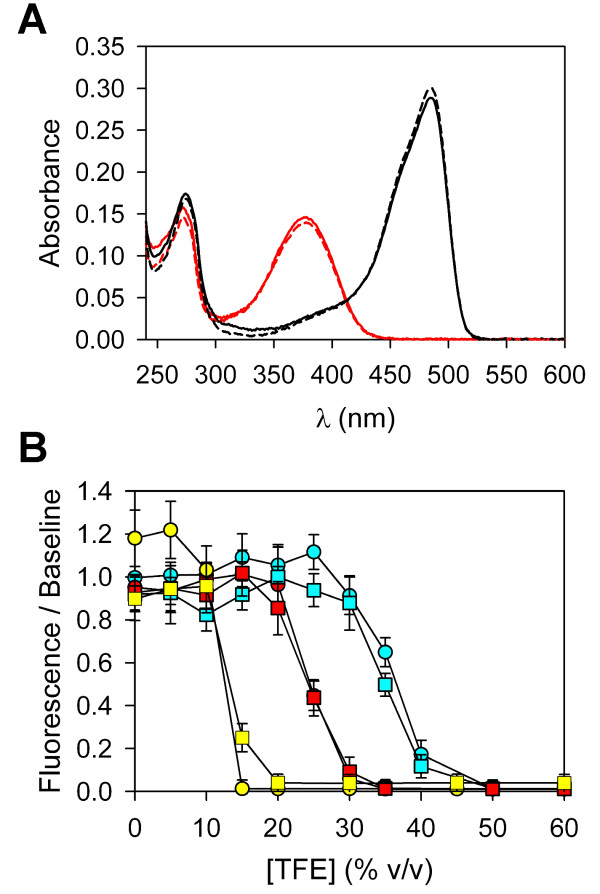
**Spectral properties of the αS-EGFP construct**. (A) Absorbance spectra of αS-EGFP (dashed lines) and EGFP (solid lines). Spectra are shown for 5 μM protein in 10 mM phosphoric acid (pH 2.4, red lines) and 10 mM NaPhos buffer (pH 7.5, black lines). (B) The normalized fluorescence emission from αS-EGFP (squares) and EGFP (circles) as a function of TFE concentration. The signal from 0.3 μM protein is measured after a 2.0 ± 0.5 minute incubation at 22°C (cyan symbols), after 2.0 ± 0.5 minutes at 37°C (red symbols), and after 24 ± 2 hours at 37°C (yellow symbols). The error bars reflect the standard deviations of measurements of three identical samples and baseline uncertainties.

Fluorescent protein tertiary structure can be disrupted by extremes of pH [21,22] or by the addition of moderate-to-high concentrations of TFE [23]. Loss of native tertiary structure results in loss of green fluorescence and a shift in the absorbance peak [21]. In Figure 5, we show that the spectral features of acid-denatured αS-EGFP are similar to those of EGFP alone. In addition, fluorescence loss occurs above ~10% TFE for both EGFP and αS-EGFP after incubation for > 24 hours at 37°C (Figure 5B).

**Figure 6:**
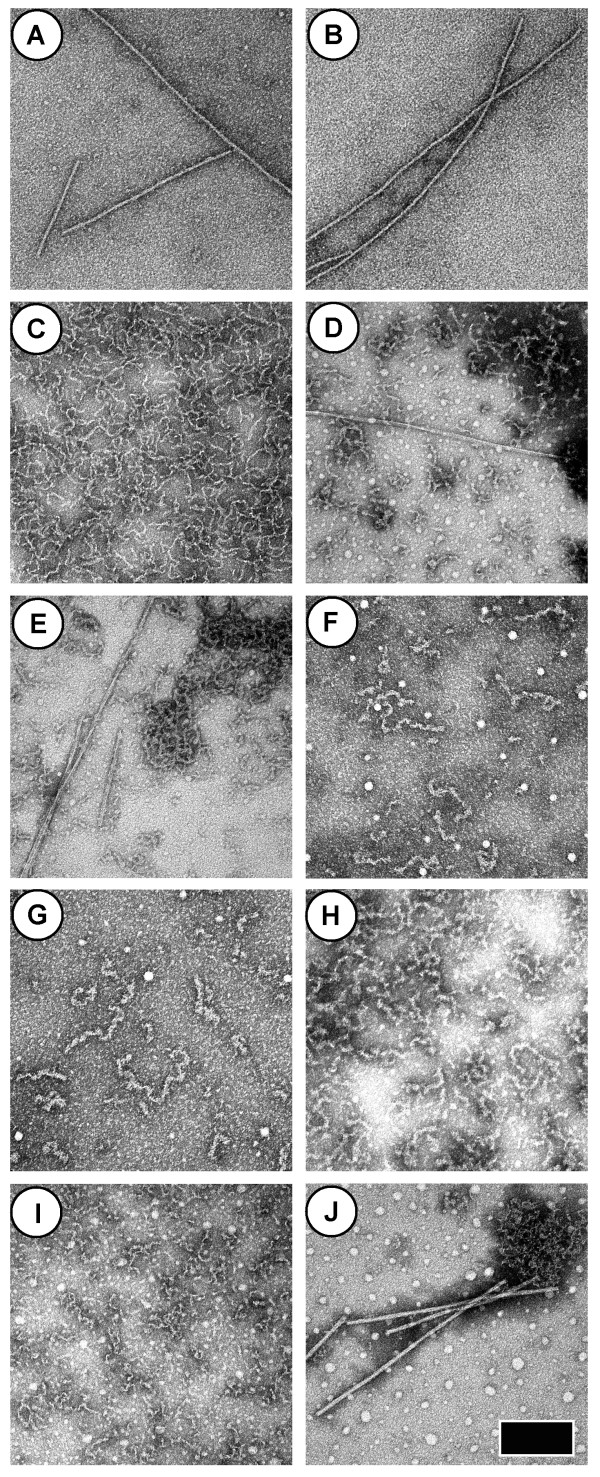
**TEM images of αS-EGFP aggregates grown in potentially denaturing conditions**. The scale bar is 200 nm wide and all images are shown at the same magnification. All samples were incubated for three weeks, and unless otherwise specified, the incubation temperatures were 37°C. The protein concentrations and other solution conditions are as follows: (A-B) 50 μM αS-EGFP at pH 2.4. (C) 50 μM αS-EGFP at pH 2.4 with 150 mM NaCl. (D-E) 50 μM αS-EGFP in pH 7.5 NaPhos with 15% TFE. (F) 50 μM αS-EGFP in pH 7.5 NaPhos with 10% TFE, after incubation at 22°C under quiescent conditions. (G) Same as F, but the solution contained 75 μM αS-EGFP. (H) Same as F, but the solution contained 15% TFE. (I) 50 μM αS-EGFP at pH 2.4 with 15% TFE. (J) 50 μM αS-EGFP at pH 2.4 with 15% TFE and 150 mM NaCl.

Figure 6 shows TEM images of αS-EGFP aggregates grown at low pH and/or in the presence of TFE. In low ionic strength, pH 2.4 solutions, rigid, amyloid-like fibrils ~12 nm in diameter were observed (Figure 6A-B). However, the inclusion of 150 mM NaCl in these solutions resulted in the formation of thin, flexible fibrils (Figure 6C). Moreover, pH 7.5 αS-EGFP solutions appear clear or cloudy-white after incubation at for > 24 hours in the presence of 10-15% TFE. TEM examination of such samples reveals a combination of amorphous aggregates, thin, flexible, fibrillar aggregates, and rigid fibrils that resemble classical amyloid (Figure 6D-E). Prolonged room-temperature, pH 7.5 incubation of αS-EGFP in the presence of 10-15% TFE resulted in the formation of flexible aggregates (Figure 6F-H). When a combination of acidic conditions and TFE were employed, both short, disordered, fibrillar aggregates and rigid fibrils were observed (Figure 6I-J).

## Discussion

In this Paper, we have demonstrated that several small extrinsic fluorescent tags are suitable for use in Aβ40 and αS aggregation studies. We show that the AMCA, TAMRA and Hilyte Fluor 488 labels do not preclude the formation of many types of amyloid and fibrillar aggregates for Aβ40 (Figure 1). These results are in accordance with previous studies of Aβ, which demonstrated that extrinsic fluorophore labelling does not prevent the formation of classic amyloid aggregates [5,7,8,10]. In addition, the Alexa Fluor 488 tag does not prevent the formation of classic amyloid fibrils and "TFE protofibrils" [19] for WT αS labelled at position 9 or position 130, and it appears that this tag is also suitable for studies of A30P and A53T αS (Figure 3).

In contrast, we find that EGFP labelling of αS favours the formation of two types of rigid aggregates when protein solutions are incubated near physiological pH (Figure 4). However, we have only been able to observe fibrils in samples that have been seeded with pre-aggregated material. Moreover, it is not clear whether the αS-EGFP fibrils retain their fluorescence; fluorescent clumps formed in samples containing αS labelled with Alexa Fluor 488, but no similar clumps were seen in the αS-EGFP samples. Recently, van Ham, et al. observed a reduction in fluorescence for fibrils formed from YFP labelled αS, which they attribute to energy migration Förster resonant energy transfer (also known as homoFRET), rather than disruption of the YFP tertiary structure [11]. It is possible that a similar effect occurs for αS labelled with EGFP tag, but we cannot verify this with our current data. Additional experiments are necessary to determine whether the native EGFP fold remains intact in the αS-EGFP fibrils.

Interestingly, we are able to grow fibrils from αS-EGFP in solutions in which EGFP tertiary structure is likely disrupted (Figure 6). Given the fact that denatured fluorescent proteins are aggregation-prone [24,25], and the observation that amyloid fibrillization may be a universal property of polypeptides [26], it is possible that the properties of the EGFP tag, rather than the smaller αS domain, dominate the fibrillization reaction under these conditions. Therefore, care must be employed when using the EGFP tag to study protein aggregation in potentially denaturing conditions.

## Conclusions

We have presented TEM images and other characterization of aggregates composed of fluorescently labelled proteins. Our images support the use of several small intrinsic fluorescent tags in studies of αS and Aβ40 aggregation. In addition, we present images of fibrils grown from αS labelled with EGFP. Near neutral pH, αS-EGFP fibrils are rigid and often feature an indistinct helix wound around a rigid core. Acid or TFE denaturation of the EGFP tag results in the formation of additional types of αS-EGFP aggregates. Our images may assist researchers in the selection of labels and in optimization of experimental conditions for protein aggregation studies involving fluorescence techniques.

## Methods

### Solutions and Reagents

MilliQ or HPLC grade water was used to prepare all solutions. Sodium phosphate (NaPhos) buffer salts were purchased from Sigma. Trizma brand pre-set pH 7.7 crystals (Sigma) were used to prepare Tris buffers that were pH ~7.4 at 37°C. Temperature-dependent changes in the pH of other buffer solutions were ignored. Acros Organics brand 99.8% pure TFE was purchased from Fisher Scientific. Sodium azide (Sigma) at ~0.02% w/v was added to all solutions incubated at ~20°C for over 24 hours. A bench-top orbital shaker operating at 200 RPM was used to agitate some samples during incubation.

### Aβ40 Solubilization

The three Aβ40 peptide tags discussed here are Hilyte Fluor 488, TAMRA (5-carboxytetramethylrhodamine), and AMCA (7-Amino-4-methylcoumarin-3-acetic acid). N-terminally fluorophore labelled, synthetic Aβ40 peptides were purchased from Anaspec. The percentage of singly-labelled Aβ40 peptide was measured by the manufacturer using HPLC and mass spectrometry, and was reported as > 90% for all batches examined in this Paper.

Protein stock solution preparation roughly followed the protocol in Bitan and Teplow [27]. Briefly, the lyophilized peptides were dissolved at ~1 mg/mL in 2 mM NaOH, and then these solutions were flash-frozen in liquid nitrogen and relyophilized. Final solubilization was accomplished by dissolving the powder into 10 mM, pH 10 carbonate buffer and filtering through YM-30 or YM-50 Microcon filters (Millipore). An exception to this procedure is shown in Figure 1A; for this sample, 0.1 mg of Hilyte Fluor 488 labelled Aβ40 was dissolved directly into water.

### Alexa Fluor 488 Labelled αS Preparation

Alexa Fluor 488 was purchased from Invitrogen, and labelling was generously performed by Trudy Ramlall and Prof. David Eliezer of Weill Cornell Medical College using previously described procedures [28]. However, serine-to-cysteine mutations and labelling at position 9 were performed on A30P and A53T, in addition to WT αS. Furthermore, C-terminal labelling of WT and A30P αS was also investigated via a glutamate-to-cysteine mutation at position 130. Free dye was removed from the samples by dialysis vs. 10 mM pH 7.5 NaPhos buffer using Slide-A-Lyzer 10,000 MW cut-off dialysis cassettes (Thermo-Fisher Scientific).

We estimated the dye-to protein ratio (*F/P*) of our dialyzed protein stocks using UV and visible light absorbance measurements via a double-beam Cary-300 spectrophotometer (Varian). All measurements were obtained using a 1 cm path length, and the *F/P *ratio was then calculated using [29]:

(1) $$\left(F∕P\right)=A^{Max}\varepsilon_{P}^{280}∕\left[\varepsilon_{F}^{Max}\left(A^{280}-CL\cdot A^{Max}\right)\right]$$

where *A^Max ^*is the measured peak absorbance, *A^280 ^*is the measured absorbance at 280 nm, $\varepsilon_{F}^{Max}$ is the dye's molar extinction coefficient at the peak, $\varepsilon_{P}^{280}$ is the molar extinction coefficient for unlabeled protein at 280 nm, and *CL *is a correction factor that accounts for the contribution of the fluorophore to the absorbance at 280 nm. We used $\varepsilon_{P}^{280}=5,120\;{\text{M}}^{\text{ - 1}}\text{c}{\text{m}}^{\text{ - 1}}$ for αS [30], while the values of *CL *and $\varepsilon_{F}^{Max}$ for Alexa Fluor 488 (0.11 and 72,000 M^-1^cm^-1^, respectively) were provided by Invitrogen.

Using equation 1, we obtained *F/P *estimates ranging from 0.9 to 2.1 for our αS stock solutions (the mean value for the nine stock solutions we prepared was 1.4, and the standard deviation was 0.4). Because αS contains no cysteine residues, excepting the one introduced for labelling purposes, the maximum *F/P *ratio should be 1 after dialysis. However, the fluorescence properties of Alexa Fluor 488 are sensitive to solution conditions and to details of the target protein sequence [31], and so the estimate provided by Equation 1 is somewhat uncertain. Therefore, our *F/P *values suggest that the labelling efficiency is reasonably good, but we cannot report a precise degree of labelling.

### αS-EGFP Expression

Vectors for mammalian expression of the human WT αS-EGFP construct were a kind gift from Prof. Bradley Hyman of Massachusetts General Hospital Medical School at Harvard University; information about this construct can be found in McLean, et al. [15]. Transformation into a bacterial vector and subsequent protein expression was performed by Dr. Cynthia Kinsland and the Cornell University Life Sciences Core Laboratories Center Protein Production Facility.

Plasmid DNA was purified with the Qiagen Miniprep kit. *E. coli *strain MachI (Invitrogen) was used as a recipient for transformations during plasmid construction and for plasmid propagation and storage. PCR was performed with Phusion DNA polymerase (New England Biolabs) per the manufacturer's instructions. DNA oligonucleotides were ordered from IDT DNA. Site-directed mutagenesis was performed by a standard PCR protocol using PfuTurbo DNA polymerase per the manufacturer's instructions (Agilent) and DpnI (New England Biolabs) to digest the methylated parental DNA prior to transformation.

Site-directed mutagenesis was performed on the provided plasmid to introduce a 6xHisTag at the C-terminus of the αS-EGFP fusion protein. The primers used for mutagenesis were: 5'-GGC ATG GAC GAG CTG TAC AAG CAC CAT CAC CAC CAT CAC-3' and 5'-CTA GAG TCG CGG CCG CTT TAG TGA TGG TGG TGA TGG TGC TT-3'. After transformation, colonies were screened for the presence of the HisTag by PCR using the following primer pair: 5'-GGG ATC CAT CGC CAC CAT GG-3' and 5'-CGC GGC CGC TTT AGT GAT GG-3'. A plasmid which screened correctly was verified by sequencing. The final construct was based on the cloning vector EGFP-N3, with αS fused to the N-terminus of EGFP and a 6xHisTag fused to the C-terminus of EGFP.

The fusion construct described above was moved into a vector for *E. coli *expression by using the following primer pair: 5'-GGG TAG CAT ATG GAT GTA TTC ATG AAA GGA CTT TC-3' and 5'-CCC TAC TCG AGT TAG TGA TGG TGG TGA TGG TGC-3'. Following amplification, the PCR product was digested with *Nde*I and *Xho*I and ligated into a similarly digested pTHT vector, resulting in an additional 6xHisTag added to the N-terminus of the total fusion construct. pTHT is a homemade vector which is equivalent to pET-28 (Novagen) with a TEV protease recognition site in place of the thrombin recognition site.

Plasmids were transformed into BL21Star (DE3) cells (Stratagene) harbouring the pRARE2 plasmid (Novagen) and selected on kanamycin/chloramphenicol media at all stages. Protein expression in shake flasks was performed as described in the pET-system manual, with induction by IPTG (1 mM) at reduced temperature (15°C) and overnight incubation post-induction. Cells were harvested by centrifugation, lysed by sonication, and HisTagged protein was purified on 5 mL HisTrap HP columns (GE) using an AKTA FPLC. Buffers used for purification were A) Binding: 20 mM Tris, pH 8.0, 500 mM NaCl, 30 mM Imidazole. B) Elution: 20 mM Tris, pH 8.0, 500 mM NaCl, 500 mM imidazole. The column was washed with A until the *A^280 ^*had returned to baseline and was then washed with 10% B in A and 15% B in A. For both washes, the wash was continued until the baseline had stabilized (several column volumes). The protein was then eluted in 100% B.

### αS-EGFP Dialysis and Buffer Exchange

To prepare the samples shown in Figure 4, which were buffered with Tris containing 100 mM NaCl or PBS (10 mM pH 7.5 NaPhos with 150 mM NaCl), the eluted protein was dialyzed into the buffer using 10,000 MWCO Slide-A-Lyzer cassettes (Pierce). When dialyzed into PBS, the protein partially precipitated, and visible white material was removed from these solutions by centrifugation for 30 minutes at 13,000 × g. The pellet was collected and used to "seed" some samples (e.g. Figure 4I-J). Some aliquots of the dialyzed protein solutions were spin-filtered using YM-100 Microcon filters (Millipore) in order to obtain mostly monomeric stock solutions. When necessary, filtered solutions were concentrated using Amicon YM-10 filters (Millipore).

For the samples shown in Figure 6, filtering was performed using a 0.22 μm syringe filter (Millex-GV, Millipore), followed by filtering with YM-100 Microcon filters (Millipore). Buffer exchange into water was performed using Amicon YM-10 filters (Millipore), and the αS-EGFP stocks were diluted into buffer or acid prior to incubation.

### Fluorescence Spectroscopy

Fluorescence emission spectra for 480-580 nm were collected using 460 nm excitation via a QuantaMaster fluorescence spectrofluorometer (Photon Technology International). All fluorescence emission signals were normalized to the emission signal from EGFP in PBS (10 mM NaPhos, 150 mM NaCl) at room temperature (22 ± 3°C). Correction for lamp fluctuations was automated by the vendor-supplied software. For the 37°C samples, the temperature was maintained during fluorescence measurements using a NesLab Endocal RTE-110 chiller/circulator (Thermo Scientific). In each case, three identical samples were measured and their standard deviations calculated in order to determine the measurement variability.

### Determination of Protein Concentrations

UV or visible light absorbance measurements via a double-beam Cary-300 spectrophotometer (Varian) were used to quantify the amount of protein in the stock solutions. The absorbance of the fluorophore was measured and concentration calculations were performed using manufacturer-supplied extinction coefficients for the organic dyes and ε = 55,000 M^-1 ^cm^-1 ^for EGFP [32]. The fluorophore and protein concentrations were assumed to be the same as in all cases.

### Transmission Electron Microscopy Imaging

The general procedure for the TEM sample preparation and imaging is described in Anderson, et al. [19]. Slight variations of these techniques were employed to obtain some of the images, including the occasional use of homemade butvar grids (both carbon-coated and uncoated butvar grids were employed), and the rare use of 1% (w/v) uranyl acetate, rather than 2% (w/v) phosphotungstic acid, stain. These differences in methodology did not significantly affect the imaging results.

### Two-Photon Action Cross-Section Measurements

A pulsed titanium sapphire Mai Tai laser (Spectra Physics) was used to excite the fluorophores over the wavelength range of 760-1000 nm. The excitation and emission light was focused through a 63x, 1.2 NA water immersion C-Apochromat objective lens (Zeiss) into ~100 nM samples, which were mounted on an inverted microscope (IX71, Olympus). The intensity of the excitation beam was measured using a photodiode, while the intensity of the emitted fluorescence was detected using a gallium arsenide phosphide photomultiplier tube (Hamamatsu). Linear fitting to the emitted light vs. incident intensity squared curves was performed at each measured wavelength and the resultant slopes were normalized to the values for a pH 11 Fluorescein standard [33] in order to determine the two-photon action cross-section for the unknown fluorophores.

## Authors' contributions

VLA and WWW designed research and analyzed data. VLA carried out all experiments and wrote the paper. All authors read and approved the final manuscript.
